# Mechanisms of Enzyme-Catalyzed Reduction of Two Carcinogenic Nitro-Aromatics, 3-Nitrobenzanthrone and Aristolochic Acid I: Experimental and Theoretical Approaches

**DOI:** 10.3390/ijms150610271

**Published:** 2014-06-10

**Authors:** Marie Stiborová, Eva Frei, Heinz H. Schmeiser, Volker M. Arlt, Václav Martínek

**Affiliations:** 1 Department of Biochemistry, Faculty of Science, Charles University, Hlavova 2030, CZ-12843, Prague 2, Czech Republic; E-Mail: vacmar@natur.cuni.cz; 2 Division of Preventive Oncology, National Center for Tumor Diseases, German Cancer Research Center (DKFZ), Im Neuenheimer Feld 280, 69120 Heidelberg, Germany; E-Mail: eva.frei@nct-heidelberg.de; 3 Radiopharmaceutical Chemistry E030, German Cancer Research Center (DKFZ), Im Neuenheimer Feld 280, 69120 Heidelberg, Germany; E-Mail: h.schmeiser@dkfz-heidelberg.de; 4 Analytical and Environmental Sciences Division, MRC-PHE Centre for Environmental & Health, King’s College London, 150 Stamford Street, London SE1 9NH, UK; E-Mail: volker.arlt@kcl.ac.uk

**Keywords:** nitro-aromatics, 3-nitrobenzanthrone, aristolochic acid I, DNA adducts, molecular modeling, NAD(P)H:quinone oxidoreductase, cytochrome P450

## Abstract

This review summarizes the results found in studies investigating the enzymatic activation of two genotoxic nitro-aromatics, an environmental pollutant and carcinogen 3-nitrobenzanthrone (3-NBA) and a natural plant nephrotoxin and carcinogen aristolochic acid I (AAI), to reactive species forming covalent DNA adducts. Experimental and theoretical approaches determined the reasons why human NAD(P)H:quinone oxidoreductase (NQO1) and cytochromes P450 (CYP) 1A1 and 1A2 have the potential to reductively activate both nitro-aromatics. The results also contributed to the elucidation of the molecular mechanisms of these reactions. The contribution of conjugation enzymes such as *N*,*O*-acetyltransferases (NATs) and sulfotransferases (SULTs) to the activation of 3-NBA and AAI was also examined. The results indicated differences in the abilities of 3-NBA and AAI metabolites to be further activated by these conjugation enzymes. The formation of DNA adducts generated by both carcinogens during their reductive activation by the NOQ1 and CYP1A1/2 enzymes was investigated with pure enzymes, enzymes present in subcellular cytosolic and microsomal fractions, selective inhibitors, and animal models (including knock-out and humanized animals). For the theoretical approaches, flexible *in silico* docking methods as well as *ab initio* calculations were employed. The results summarized in this review demonstrate that a combination of experimental and theoretical approaches is a useful tool to study the enzyme-mediated reaction mechanisms of 3-NBA and AAI reduction.

## 1. Introduction

Nitro-aromatics such as nitro-polycyclic aromatic hydrocarbons (nitro-PAHs) are widely distributed environmental pollutants found in exhausts from diesel and gasoline engines and on the surface of ambient air particulate matter [1,2,3,4]. The major source of these compounds is incomplete combustion of the compounds present in diesel. However, they are also formed by reactions of PAHs with nitrogen oxides in the atmosphere [5,6]. The increased lung cancer risk after exposure to environmental nitro-PAHs and their detection in the lungs of non-smokers with lung cancer has led to considerable interest in assessing their potential cancer risk [1,2,7,8]. In contrast to these nitro-pollutants, occurrence of nitro-aromatic compounds with a natural origin is rare [9,10,11]. Both groups of nitro-aromatics have become of enormous concern because of their mutagenicity and carcinogenicity [1,2,10,11,12].

**Figure 1:**
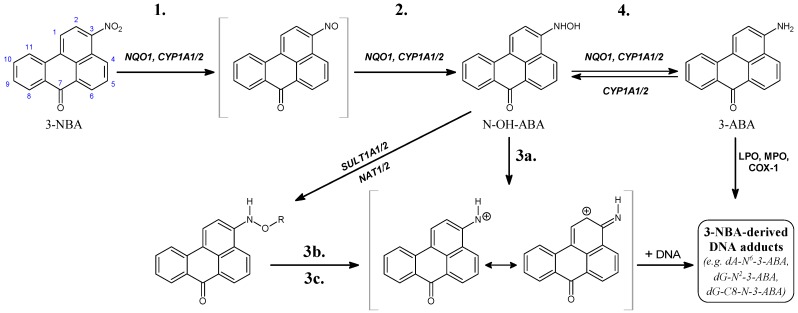
Pathways of metabolic activation and DNA adduct formation of 3-nitrobenzanthrone (3-NBA) and 3-aminobenzanthrone (3-ABA). See text for details. *N*-OH-ABA, *N*-hydroxy-3-aminobenzanthrone; NQO1, NAD(P)H: quinone oxidoreductase; NAT, *N*,*O*-acetyltransferases; SULT, sulfotransferase; COX-1, cyclooxygenase 1; CYP, cytochrome P450; LPO, lactoperoxidase; MPO, myeloperoxidase; POR, NADPH:CYP oxidoreductase; R = –COCH_3_ or –SO_3_H; dA-*N*^6^-3-ABA, 2-(2'-deoxyadenosin-*N*^6^-yl)-3-aminobenzanthrone; dG-*N*^2^-3-ABA, *N*-(2'-deoxyguanosin-*N*^2^-yl)-3-aminobenzanthrone; dG-C8-*N*-3-ABA, *N*-(2'-deoxyguanosin-8-yl)-3-aminobenzanthrone. Individual reaction steps are assigned by numbers 1–4 (see also Table 3).

**Figure 2:**
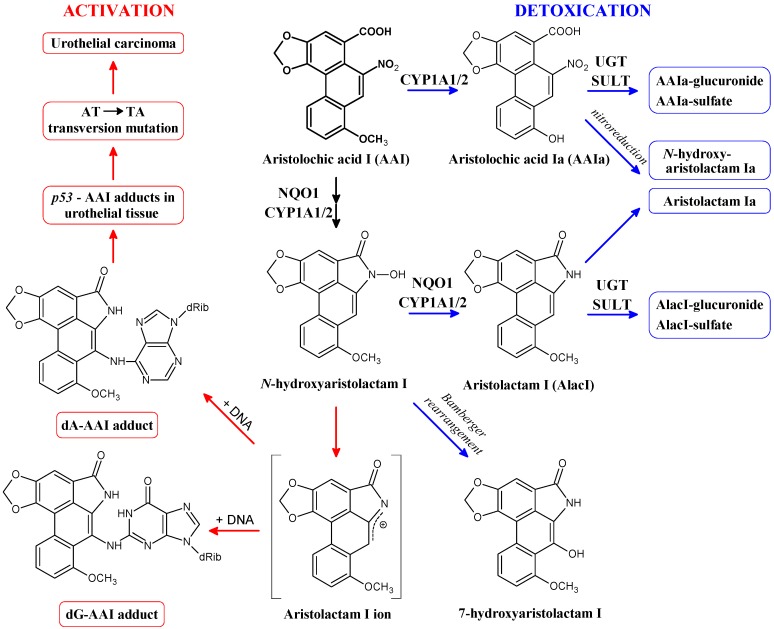
Activation and detoxication pathways of aristolochic acid I (AAI). dA-AAI, 7-(deoxyadenosin-*N*^6^-yl)aristolactam I; dG-AAI, 7-(deoxyguanosin-*N*^2^-yl)aristolactam I; CYP1A1/2, cytochrome P450 1A1 and 1A2; NQO1, NAD(P)H:quinone oxidoreductase; UGT, UDP glucuronosyltransferase; SULT, sulfotransferase.

3-Nitrobenzanthrone (3-NBA; 3-nitro-7*H*-benz[*de*]anthracen-7-one; (Figure 1) and aristolochic acid I (AAI; 8-methoxy-6-nitro-phenanthro-(3,4-*d*)-1,3-dioxolo-5-carboxylic acid; (Figure 2) as environmental and natural (plant) nitro-aromatics, respectively, were thoroughly investigated because of their strong mutagenicity and carcinogenicity [1,2,10,11].

The nitro-ketone 3-NBA (Figure 1) occurs in diesel exhaust and in airborne particulate matter [13,14,15,16]. 3-NBA exhibits an extremely high mutagenic activity [13,15,17] and is also a genotoxic carcinogen causing lung tumours in rats [15,16]. The major DNA adducts formed by 3-NBA both *in vitro* and *in vivo* are derived from nitroreduction and have been structurally identified as 2-(2'-deoxyguanosin-*N*^2^-yl)-3-aminobenzanthrone (dG-*N*^2^-3-ABA) and *N*-(2'-deoxyguanosin-8-yl)-3-aminobenzanthrone (dG-C8-*N*-3-ABA) [15,18,19,20]. These adducts are most probably responsible for the formation of G to T transversion mutations induced by 3-NBA *in vitro* and *in vivo* [21,22,23]. 3-NBA has been classified as possible human carcinogen (Group 2B) by the International Agency for Research on Cancer (IARC) [2].

The herbal drug aristolochic acid (AA), a natural mixture of 8-methoxy-6-nitro-phenanthro-(3,4-*d*)-1,3-dioxolo-5-carboxylic acid (AAI; Figure 2) and 6-nitro-phenanthro-(3,4-*d*)-1,3-dioxolo-5-carboxylic acid (AAII), is derived from *Aristolochia* species and the cause of aristolochic acid nephropathy (AAN) [24,25,26,27,28]. AAN is a rapidly progressive renal fibrosis with a high risk for the patients of developing upper urothelial tract carcinoma and, subsequently, bladder urothelial carcinoma [28,29]. Exposure to AA has also been linked to inhabitants of rural areas in the Balkans who develop nephropathy—Balkan endemic nephropathy (BEN) [28,30,31,32,33,34].

Exposure of experimental animals to AA leads to characteristic AA-DNA adducts in renal tissue after reductive activation. The same DNA adducts, mainly 7-(deoxyadenosin-*N*^6^-yl)aristolactam I (dA-AAI) (Figure 2), were detected in kidneys of AAN and BEN patients whereby their exposure to AA was identified [29,30,32,35,36,37,38]. This deoxyadenosine adduct causes characteristic AT-TA transversions in critical genes of oncogenesis such as tumor suppressor *TP53* (Figure 2) and AT-TA mutations have indeed been found in urothelial tumours of AAN patients [31,32,33,39,40,41,42]. This indicates the molecular mechanism associated with AA-induced carcinogenesis [31,38,43]. AA has been classified as a Group I human carcinogen by IARC [10,11].

Previous results demonstrated that both nitro-aromatics are genotoxic mutagens and carcinogens after metabolic activation (for a review see [15,25,27,44,45,46,47]). Therefore, the identification of the enzymes responsible for their activation is of great importance.

This review summarizes the latest findings that identified enzymes catalyzing the reductive activation of 3-NBA, and the major toxic component of the AA natural plant extract, AAI. Moreover, the molecular mechanisms of the reactions leading to the reductive activation of 3-NBA and AAI *in vitro* and *in vivo* are identified from the results summarized in this review. The data shown also shed light on the different efficacies of conjugation enzymes to metabolically activate both nitro-compounds. The results presented in this review demonstrate that a combination of experimental and theoretical approaches is useful to resolve the enzyme-mediated reaction mechanisms responsible for the genotoxicity of both nitro-aromatic carcinogens.

## 2. Cytosolic NAD(P)H:Quinone Oxidoreductase (NQO1) and Microsomal Cytochrome P450 (CYP) 1A1 and 1A2 Enzymes Reductively Activate 3-NBA and AAI

Using a combination of multiple *in vitro*, *in vivo* and *ex vivo* approaches employing pure and recombinant enzymes, subcellular fractions (microsomes and cytosols), selective enzyme inhibitors and rodent models (including knock-out and humanized animals) we identified that 3-NBA and AAI are most efficiently activated by cytosolic NQO1 to species forming DNA adducts but CYP1A1/2 have also been shown to be capable of reductively activating both 3-NBA and AAI.

### 2.1. Reductive Activation of 3-NBA and Aristolochic Acid I (AAI) by Human NQO1

Human NQO1 in the presence of its cofactor, NADPH, reductively activates 3-NBA to the reactive intermediate *N*-hydroxy-3-aminobenzanthrone (*N*-OH-3-ABA) (see reaction step 2 in Figure 1) that dissociates to a ion that exhibits both nitrenium and carbenium character because the positive charge is delocalized to both nitrogen and carbon sites (step 3a in Figure 1), forming DNA adducts both *in vitro* and *in vivo* (Figure 1 and Figure 2; Table 1) [15,21,48,49,50,51,52,53]. *N*-OH-3-ABA can be further reduced to 3-aminobenzanthrone (3-ABA; step 4 in Figure 1) [14,51,52,54]. Of the 3-NBA-derived DNA adducts generated by NQO1, three major adducts were identified as 2-(2'-deoxyadenosin-*N*^6^-yl)-3-aminobenzanthrone (dA-*N*^6^-3-ABA), dG-*N*^2^-3-ABA and dG-C8-*N*-3-ABA [16,20,55,56,57,58] (Figure 1). Interestingly, 3-NBA and its amino metabolite, 3-ABA, are potent inducers of NQO1 in liver, lung and kidney of rats. Such induction finally resulted in a more than 10-fold increase in the levels of 3-NBA-DNA adducts in *ex vivo* incubations of 3-NBA with DNA and liver, kidney and lung cytosols isolated from these animals [50,51,53], emphasizing the importance of NQO1 in the genotoxicity of 3-NBA.

NQO1 also activates AAI to a reductive metabolite *N*-hydroxyaristolactam I [59,60] that dissociates to a cyclic ion capable of binding to the exocyclic amino groups of purine bases forming DNA adducts (for a review see [25,27,44,45,46,47]). Three major adducts are formed during this reaction and were identified as 7-(deoxyguanosin-*N*^2^-yl)aristolactam I (dG-AAI), dA-AAI and 7-(deoxyadenosin-*N*^6^-yl) aristolactam II (dA-AAII) (Figure 2). *N*-hydroxyaristolactam I can be further reduced to aristolactam I or rearrange to 7-hydroxyaristolochic acid I (Figure 2) [25,27,44,45,46,60]. AAI also induces NQO1 in kidney, the target organ for AAI toxicity, of mice and rats treated with this carcinogen [61,62,63,64,65]. This induction leads to up to 2.5-fold higher AAI-DNA adduct levels in *ex vivo* incubations of AAI with DNA and renal cytosols isolated from these rodents [62,65]. This finding underlined the significance of NQO1 in the formation of AAI-DNA adducts.

Dicoumarol, an inhibitor of NQO1 [66,67], reduces 3-NBA- and AAI-DNA adduct formation catalyzed by human NQO1 *in vitro* by almost 99% (Table 1). These results not only confirm the efficiency of NQO1 to activate 3-NBA and AAI, but also proved the potency of dicoumarol as an NQO1 inhibitor *in vitro*. Similarly, a major role of NQO1 in the activation of both nitro-aromatics was found in human hepatic and renal cytosolic fractions. The formation of 3-NBA- and AAI-DNA adducts in these subcellular fractions was dependent on the presence of the cofactor of NQO1, NADPH, and again dicoumarol was shown to be a potent inhibitor [48,52,68,69]. Recently, this NQO1 inhibitor was also utilized to study the role of NQO1 in AAI activation *in vivo* [62,70].

### 2.2. Participation of Conjugation Enzymes in Activation of 3-NBA and AAI

In human hepatic cytosols containing NQO1 and its cofactor NADPH, addition of the *N*,*O*-acetyltransferase (NAT) and sulfotransferase (SULT) cofactors, acetyl-CoA and 3'-phospho-5'-adenosine-phospho-sulfate (PAPS), respectively, significantly increased the formation of 3-NBA-DNA adducts [48]. This was confirmed in incubations with recombinant enzymes (NAT1, NAT2, SULT1A1 and SULT1A2), where NAT2 was shown to be most effective (Table 1) [52]. A role of these conjugating enzymes in 3-NBA genotoxicity was also demonstrated in genetically-modified V79 cells overexpressing these transferases [71,72]. The importance of NAT2 in the overall capacity of human hepatic cytosols to activate 3-NBA was corroborated by correlation analyses [48]. Whereas no correlation was observed between the activities of NQO1, SULTs or NAT1 and 3-NBA-DNA adduct levels in cytosolic samples from several human donors, highly significant correlations were observed between the activity of NAT2 and 3-NBA-derived DNA adduct formation (*r* = 0.73, *p* < 0.01) [48].

**Table 1:** DNA adduct formation by 1 μM 3-NBA and AAI activated by human NQO1 and NATs (NAT1/2) and SULTs (SULT1A1/2).

Enzymatic System	Total Levels of DNA Adducts in RAL ^a^ (Mean ± SD/10^8^ Nucleotides)	
	3-NBA	AAI
NQO1 + NADPH	10 ± 2	15 ± 2
NQO1 + NADPH + dicoumarol	0.1 ± 0.01	0.4 ± 0.04
NQO1 + NADPH + SULT1A1 + PAPS ^b^	16 ± 2	15 ± 2
NQO1 + NADPH + SULT1A2 + PAPS	36 ± 2	16 ± 2
NQO1 + NADPH + NAT1 + acetyl-CoA ^c^	16 ± 2	15 ± 2
NQO1 + NADPH + NAT2 + acetyl-CoA	1720 ± 50	17 ± 2

^a^, RAL, relative adduct labeling; Results are presented as means ± SD of triplicate *in-vitro* incubations. Experimental conditions are as described [52,69]; ^b^, 0.4 mM PAPS; ^c^, 2 mM acetyl-CoA

In contrast, the contribution of these conjugation enzymes to the activation of 3-NBA, NAT1, NAT2, SULT1A1 and SULT1A2 was ineffective to influence AAI-DNA adduct formation mediated by NQO1 (Table 1) [69,73]. Further, similar results were found in human hepatic and renal cytosols. These findings were in line with a previous study utilizing cytosolic samples from several human donors to identify the participation of cytosolic enzymes involved in AAI activation [68]. Using correlation analyses (*r* = 0.85; *p* < 0.001), only the participation of NQO1 was found to be highly significant for AAI-DNA adduct formation *in vitro*. Collectively, these findings indicate that NATs and SULTs do not play a significant role in the bioactivation of AAI in human liver and kidney. However, it is noteworthy that Meinl *et al.* [74] found that the expression of human SULT1A1 in bacterial and mammalian cells enhances the mutagenic activity of the natural plant extract AA. Therefore, it is possible that in cellular systems overexpression of human SULT1A1 might contribute to AAI genotoxicity. In addition, SULT1A1-humanized animal models would help to investigate the contribution of this SULT isoenzyme to AAI activation *in vivo*.

### 2.3. Reductive Activation of 3-NBA and AAI by Human CYP1A1 and 1A2

Besides cytosolic NQO1, microsomal enzymes such as NADPH:CYP oxidoreductase (POR) and/or CYP enzymes are able to metabolically activate 3-NBA and AAI by simple nitroreduction [75,76,77,78,79].

A role of POR in the bioactivation of 3-NBA *in vivo* was evaluated by treating Hepatic Reductase Null (HRN) mice intraperitoneally with 3-NBA [48]. In HRN mice POR, the electron donor of CYP enzymes is specifically deleted in hepatocytes resulting in essentially no hepatic CYP function. The results of this study indicated that *in vivo* 3-NBA is predominantly activated by cytosolic NQO1 rather than microsomal POR [48]. However, participation of POR and/or other microsomal enzymes such as CYP enzymes in 3-NBA-DNA adduct formation cannot be excluded. Whereas human recombinant CYPs such as CYP1A1, 1A2, 2B6 and 2D6 were found to efficiently activate 3-NBA under anaerobic conditions to form DNA adducts, POR and other CYPs such as CYP2A6, 2C9, 2E1 or 3A4 were less effective or ineffective. As shown in Table 2, CYP1B1 activated 3-NBA even less efficiently than POR [79]. Contribution of human CYP1A2 to 3-NBA-DNA adduct formation was also found in V79 cells, where this CYP was overexpressed [72]. Of the CYP enzymes reducing 3-NBA, CYP1A1 and 1A2 were also found to oxidize 3-ABA to *N*-OH-3-ABA dissociating to the ion binding to DNA (Figure 1) [80]. In addition, CYP1A1 and 1A2 are induced in liver, lung and kidney of rats not only treated with 3-NBA, but also with 3-ABA [49,50,51,53]. DNA adduct levels increased up to 12-fold due to the induction of CYP1A1/2 in lung, kidney and liver in 3-NBA- or 3-ABA-treated rats using different routes of administration including intratracheal instillation of 3-NBA [49,50,51,53]. These enzyme inductions by 3-NBA and 3-ABA seem to produce concerted regulatory effects of these compounds on their own metabolism. Therefore, both CYP1A1 and 1A2 are important enzymes contributing both to reductive activation of 3-NBA and the oxidative activation of 3-ABA leading to *N*-OH-3-ABA in both cases. To shed light on the reaction mechanisms of 3-NBA reduction by CYP1A1/2, *in silico* docking of 3-NBA to the active sites of the CYP1A1/2 enzymes was performed in the present work (see Section 4.2.).

**Table 2:** DNA-adduct formation by 300 μM 3-NBA and AAI activated by human recombinant POR, CYP1A1, 1A2 and 1B1.

Enzymatic System	Total Levels of DNA Adducts in RAL ^a^ (Mean ± SD/10^8^ Nucleotides)	
	3-NBA	AAI
POR + NADPH	10 ± 2	15 ± 2
CYP1A1 + POR + NADPH	52 ± 5	84 ± 7
CYP1A2 + POR + NADPH	48 ± 5	126 ± 12
CYP1B1 + POR + NADPH	5 ± 1	11 ± 1

^a^, RAL, relative adduct labeling; Results are presented as means ± SD of triplicate *in-vitro* incubations. Experimental conditions are as described [77,78,79].

Microsomal POR plays only a minor role in the reductive activation of AAI [75,77,78]. In human and rat hepatic microsomes, most of the activation of AAI is attributable to its reduction by CYP1A2, and to lesser extent by CYP1A1 [75,77,78]. These results were confirmed by using selective inhibitors of POR or CYP1A1 and 1A2, the recombinant human enzymes and correlation analyses. Whereas an inhibitor of POR, α-lipoic acid [81], did not inhibit AAI-DNA adduct formation in the *ex vivo* incubations of AAI and DNA with human hepatic microsomes, inhibitors of CYP1A1/2 and 1A2, α-naphthoflavone and furafylline, respectively, significantly decreased levels of AAI-DNA adducts in these incubations [75,77]. Under anaerobic conditions, significantly higher levels of AAI-derived DNA adducts were generated by CYP1A1/2 than by human POR or other tested human CYPs (CYP1B1, 2A6, 2B6, 2C9, 2D6, 2E1, 3A4 or 3A5) (see Table 2) [77]. In addition, total DNA binding by AAI was highly significantly correlated with 7-ethoxyresorufin *O*-deethylase activity, a marker for CYP1A1/2 (*r* = 1.0; *p* < 0.001). Since CYP1A1 is expressed in human liver at relatively low levels [82,83], its contribution to AAI activation in human liver is much lower than that of CYP1A2. However, CYP1A1 is important in AAI activation in the kidney as it is expressed there and even induced by AAI itself [62,75,77].

A recent study utilized two *CYP1A*-humanized mouse lines (*hCYP1A1_1A2_Cyp1a1/1a2^−/−^*_*Ahr^b1^* and *hCYP1A1_1A2_Cyp1a1/1a2^−/−^*_*Ahr^d^*) that carried functional human *CYP1A1* and *CYP1A2* genes and lacked the mouse orthologous genes [84,85]. This study showed that human CYP1A1 and 1A2 are also responsible for AAI activation to form DNA adducts *in vivo* [86]. AAI-induced DNA adduct levels were higher in *CYP1A*-humanized mice (predominantly in kidney and liver) relative to wild-type mice, indicating that expression of human CYP1A1 and 1A2 in mice leads to higher AAI bioactivation than in mice containing the mouse CYP1A1 and 1A2 orthologs [86]. This finding underlines the importance of CYP1A1/2 enzymes in the reductive activation of AAI in humans. A role of both CYPs in AAI metabolism was also demonstrated in *Cyp1a1^−/−^* and *Cyp1a2^−/−^* single-knockout and *Cyp1a1/1a2^−/−^* double-knockout mouse lines [87].

CYP1A1 is expressed in human kidney and thus can contribute to AAI bioactivation in this organ. However, its expression levels are low (<0.005 pmol CYP1A1/mg protein) [77,88], indicating that the overall impact of this enzyme on AAI activation in this organ seems to be low. CYP3A5 which is also expressed in human kidney has recently been found to be capable of activating AAI, but less efficiently than CYP1A1 and 1A2 [89]. Hence, a contribution of CYP3A5 to AAI activation might partially explain the efficacy of microsomes isolated from a human kidney to activate AAI used in a previous study [77]. In this study, the efficiency of the renal microsomes to activate AAI was comparable to that of microsomes isolated from human livers.

However, it should be emphasized that CYP1A1/2 play a dual role in AAI metabolism. Whereas under anaerobic (*i.e.*, reductive) conditions human CYP1A1 and 1A2 [86] can bind AAI as a ligand and reduce this carcinogen (see Section 4.2) [75,77,78,90], under aerobic (*i.e.*, oxidative) conditions both CYP enzymes are able to *O*-demethylate AAI to a detoxification metabolite, 8-hydroxyaristolochic acid I (aristolochic acid Ia, AAIa; Figure 2). Under these (aerobic) conditions, AAI behaves as a classical substrate of human CYP1A1 and 1A2, where one oxygen atom is utilized for *O*-demethylation of AAI to form AAIa [46,47,62,86,87,89,91,92,93]. These experimental results strongly suggest that, besides CYP1A1 and 1A2 expression levels, the oxygen level in tissues affects the balance between AAI nitroreductive activation and oxidative detoxication by these CYPs *in vivo* [47,62,86,87,89]. This suggestion is further supported by theoretical approaches as discussed in Section 3 and Section 4 [46,90]. Additional studies are needed to resolve which of these two opposite reactions (reduction *versus* oxidation) catalyzed by CYP1A1/2 prevails *in vivo*.

## 3. Calculation of 3-NBA and AAI Reduction, Heterolytic Cleavage of 3-NBA and AAI *N*-Hydroxyl Derivatives and Their Sulfate or Acetate Conjugates—Thermodynamic Approaches

The efficiency of human NQO1 to reductively activate 3-NBA and AAI to form DNA adducts *in vitro* was up to 24-fold higher than that of human CYP1A1/2 (Table 1 and Table 2). Several properties of the molecules might be responsible for the potency of 3-NBA and AAI to be reduced to form DNA adducts. Amenability of 3-NBA and AAI to chemical and enzymatic reduction might be one of the reasons. *Ab initio* calculations were employed to test this hypothesis [52,69,73,94,95,96].

In the case of 3-NBA, the absolute values of reaction free energies (ΔG in Table 3) of individual reaction steps (1-reduction of 3-NBA to 3-nitroso-benzanthrone, 2-reduction of 3-nitroso-benzanthrone to *N*-OH-3-ABA, and 4-reduction of *N*-OH-3-ABA to 3-ABA in Figure 1 and Table 3) of 3-NBA reduction were calculated with two approaches, the polarizable conductor calculation model (CPCM) [95] and the Langevin dipoles model (LD) [96]. As the Δ*G*s were lower than −27 kcal/mol, these reaction steps should not be rate limiting in the presence of suitable catalysts. The dissociation of *N*-OH-3-ABA (step 3a in Figure 1) is only slightly thermodynamically favored. Therefore, the dissociation of *N*-OH-3-ABA is likely the rate limiting step, determining the overall reaction rate of the nitrenium/carbenium ion formation from 3-NBA.

**Table 3:** Standard reaction free energies of individual reaction steps of 3-NBA (**A**) and AAI (**B**) conversion calculated by quantum chemical approach in combination with the polarizable conductor calculation model (CPCM) and the Langevin dipoles model (LD) (for methods, see [52,69,73,94,95,96]).

(**A**)	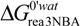 ** [kcal/mol]** ^a^					
Reaction step (Figure 1) solvation model	**1.**	**2.**	**3a.**	**3b.**	**3c.**	**4.**
	NO_2_ → NO	NO → NHOH	NHOH → NH^+^ + OH^−^	NHOAc → NH^+^ + Ac^−^	NHOSO_3_^−^ → NH^+^ + SO_4_^2−^	NHOH → NH_2_
CPCM	−46.9	−28.3	22.3	−6.1	−8.3	−64.5
LD	−44.0	−27.0	−4.5	−22.3	−24.8	−67.7
(**B**)	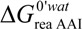 **[kcal/mol]** ^a^					
Reaction step (Figure 3) solvation model	**1.**	**2 + 3.**	**4a.**	**4b.**	**4c.**	**5.**
	NO_2_ → NO	NO → *N*-OH-lact + OH^−^	*N*-OH-lact → *N*-lact^+^ + OH^−^	*N*-OAc-lact → *N*-lact^+^ + Ac^−^	*N*-OSO_3_^−^-lact → *N*-lact^+^ + SO_4_^2−^	*N*-OH-lact → *N*-lact + H_2_O
CPCM	−46.7	−19.1	30.6	0.6	−4.9	−70.7
LD	−40.4	−9.1	−7.1	−29.4	−24.6	−69.8

^a^, The standard (biochemical) free energy change of this reaction step was corrected to pH = 7; The geometry optimizations of all reactants and products were done using *ab initio* approach implemented in Gaussian03 program suite [94]. All calculations were performed on the Hartree-Fock (HF) level of theory in conjunction with 6–31 + G(d) basis set.

**Figure 3:**
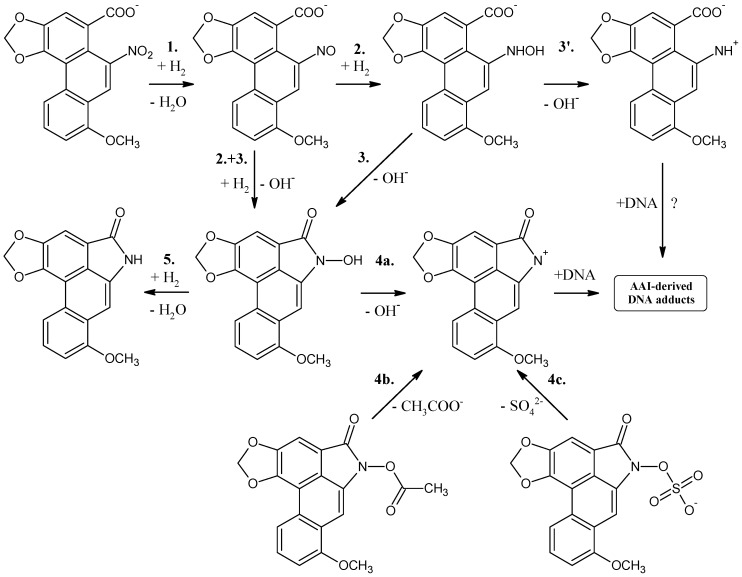
Proposed scheme of reaction steps for aristolochic acid I reduction.

The chemical structures of all AAI-DNA adducts identified indicate that a cyclic ion with a delocalized positive charge (aristolactam I ion) is the ultimate electrophilic species (see Figure 2). The reduction of the nitro group leads to the nitroso and *N*-hydroxy derivatives (steps 1, 2 and 3 in Figure 3), which are both stabilized by *peri* interaction. Condensation with the carboxylic acid moiety in *peri* position leads to a cyclic hydroxamic acid (*N*-hydroxyaristolactam I), which has been identified in the urine of AA-treated rats [60], and is expected to be the precursor of the cyclic ion with a delocalized positive charge (step 4 in Figure 3), which reacts covalently with DNA via the C7 position (Figure 2). The mechanism of *N*-hydroxyaristolactam I formation has not been elucidated, but the values of standard reaction free energies of individual reductive reaction steps suggest the above mentioned mechanism, namely condensation of the nitroso and *N*-hydroxy derivatives with the carboxylic acid moiety in *peri* position (steps 2 and 3 in Figure 3) as most probable.

Interestingly, only one type of DNA adducts is formed from AAI, in which carbon C7 of AAI is bound to either an adenine or guanine amino group in DNA [25,27,38,59]. However, it is also noteworthy that AAI forms DNA adducts with cytosine but the structure has not yet been elucidated [97,98]. The apparently preferred generation of C7 adducts by AAI, as opposed to C2 adducts by 3-NBA, suggests that the carboxylic group of AAI plays a role in controlling the regioselectivity of the reaction and the pattern of DNA adducts formed by AAI. As suggested by Priestap *et al.* [99], the ion formed from AAI might be in an ion pair in which the cation and the anion (the carboxylate group) are in the same molecule (see the compound formed by step 3' in Figure 3). The regiospecificity of DNA base attack at position C7 might be caused by the occurrence of this intramolecular ion pair that fixes the anion at one side of the reaction centre. The ion pair prevents the participation of the nitrenium nitrogen atom in reactions with nucleophiles, leaving the electron deficient position C7 as the only site for nucleophilic attack [99]. In contrast, other nitroarenes lacking the carboxylic group including 3-NBA can form DNA adducts both at nitrogen and *ortho* carbon. Indeed, besides dA-*N*^6^-3-ABA and dG-*N*^2^-3-ABA, the dG-C8-*N*-ABA adduct is also generated from the cation formed from the *N*-hydroxylated intermediate of 3-NBA, *N*-OH-3-ABA (Figure 1) [20,21,55,56].

The data obtained with the two different solvation models are quite different as soon as charged molecules are involved. The data obtained with the LC model seem more reliable than those resulting from the CPCM calculations and will be discussed below. While the *N*-OH-3-ABA is relatively stable under physiological conditions (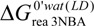 = −4.5 kcal/mol), which implies that it requires conjugation to decompose efficiently to the cation (see Table 3), the reaction free energy of dissociation of *N*-hydroxyaristolactam I found by the LD approach is −7.1 kcal/mol, which is 2.6 kcal/mol lower than for the *N*-hydroxylamine derivative of 3-NBA (Table 3; reaction step 3a for 3-NBA reduction and 4a for AAI reduction). Hence, *N*-hydroxyaristolactam I decomposes spontaneously under the same conditions. This indicates that the N-O bond in *N*-hydroxyaristolactam I is less stable than in *N*-OH-3-ABA. Consequently, if the dissociation of the N-O bond is relatively fast, it is not the rate limiting step in AAI-DNA adduct formation and any conjugation reaction would not make dissociation faster and would not lead to a higher DNA adduct level. These findings indicate that the overall rate controlling step during AAI reductive activation is not the enzymatic conjugation followed by spontaneous formation of a cyclic cation, but rather the initial nitro reduction mediated by NQO1.

Although the CPCM solvation predicts Δ*G* values of reactions 1, 2 and 4 for 3-NBA and 1 and 2 + 3 for AAI (Table 3) similar to those calculated in the LD solvation model, CPCM fails to provide realistic Δ*G*s for the reaction step 3 for 3-NBA and 4 for AAI (Table 3). Therefore, the CPCM model, widely used for evaluating the solvent effect in *ab initio* calculations [95], is less suitable to properly predict a realistic Δ*G* for this reaction step involving charged molecules. A possible explanation could be the insufficient parameterization of the CPCM solvation model implemented in Gaussian 03 for charged molecules such as ions formed from *N*-hydroxylated metabolites of 3-NBA and AAI, while parameterization of ChemSol v2.1 [100] is done also e.g., for charged amino acids. However, these results have to be carefully interpreted, because of the relatively small energy differences found in the 3-NBA and AAI cases. At this level of theory, these calculations only have a relative accuracy in excess of about ±5 kcal/mol.

Because of similarities among standard free energies of reduction of 3-NBA and AAI found in thermodynamic approaches (Table 3), the amenability of 3-NBA and AAI to chemical reduction seems not to be a major reason responsible for the differences in the reductive activation of these nitro-aromatics by NQO1 and CYP1A1/2.

## 4. Binding of 3-NBA and AAI to the Active Sites of NQO1 and CYP1A1/2 Enzymes

Another reason that might be responsible for the difference in the ability of NQO1 and CYP1A1/2 to reduce 3-NBA and AAI to species forming DNA adducts may be the affinity of these compounds to the active sites of these enzymes.

### 4.1. Binding of 3-NBA and AAI to the Active Site of NQO1

**Figure 4:**
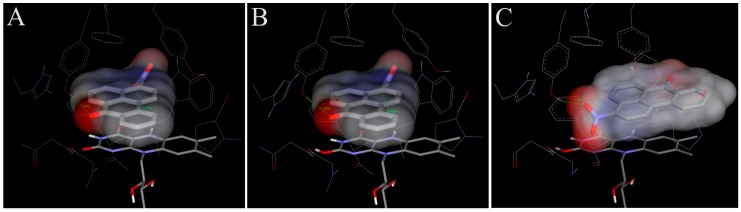
The binding orientations resulting from molecular docking calculations enabling the hydrogen transfer from N5 of isoalloxazine ring of FAD to the nitro group of 3-NBA (**A**,**B**) are shown docked to the active site of human NQO1. The two possible forms of reduced isoalloxazine ring—ionized enolate and protonated enol—bound to the NQO1 active site are labeled with subscript A and B, respectively. Binding orientations favoring a proposed alternative three-step (e^−^, H^+^, e^−^) reduction is labeled as (**C**). The 3-NBA ligand is positioned parallel to the flavin prosthetic group. FAD cofactor and amino acids residues within 5.5 Å from ligand are rendered as bold sticks, and sticks and lines, respectively. Panels A and B are taken from [52].

**Figure 5:**
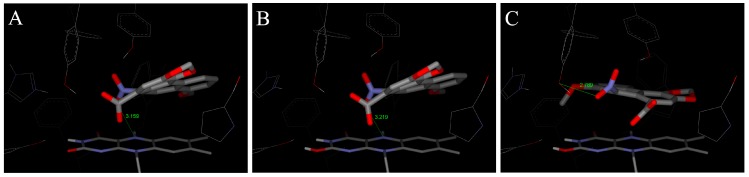
The binding orientations resulting from molecular docking calculations facilitating the hydride transfer from N5 of the isoalloxazine ring of reduced FAD of human NQO1 to the oxygen of AAI. The two possible forms of the reduced isoalloxazine ring (ionized enolate and protonated enol) bound to the NQO1 active site are labeled (**A**) and (**B**), respectively. Binding orientations favoring a proposed alternative three-step (e^−^, H^+^, e^−^) reduction is labeled as (**C**). AAI and its derivative, FAD cofactor and amino acids residues within 5.5 Å from ligand are rendered as bold sticks, and sticks and lines, respectively [69,73].

In order to examine the molecular basis of the reductive activation of 3-NBA and AAI by NQO1, their binding to the active centre of NQO1 was modeled. *In silico* docking of 3-NBA and AAI to the active site of the NQO1 dimeric molecule was performed by the soft-soft (flexible) docking procedure, which is suitable to model enzymatic processes [52,69,73]. The calculated model structures for the NQO1-3-NBA and NQO1-AAI complexes indicated that both compounds fit well into the active site of NQO1 with similar binding affinities. However, there are still insufficient mechanistic and kinetic studies on the mechanism and substrate specificity of NQO1. Based on X-ray structures of binary complexes of duroquinone and human NQO1, a two-electron reduction mechanism by direct hydride transfer from N5 of a flavin cofactor of NQO1 to the substrate has been proposed [101]. Docking calculations using the dimeric biologically active molecule of NQO1, containing either of the two possible forms of reduced flavin adenine dinucleotide (FAD)—the anionic form or the protonated enol form—predict negligible differences between 3-NBA and AAI binding affinities. The estimated free energies of 3-NBA or AAI binding to NQO1 with the anionic form of FAD or the protonated enol form are similar, 5.7 or 5.7 kcal/mol for 3-NBA, respectively, and 6.4 or 6.3 kcal/mol for AAI, respectively (Figure 4 and Figure 5, Table 4).

**Table 4:** The predicted binding free energies and distances facilitating the H-transfer for AAI, 3-NBA and their reduced metabolites to human NQO1. The data is taken from previous studies [52,69,73].

Compound	NQO1 FADH^−^ Deprotonated (Anionic Form)		NQO1 Enol-FADH_2_ (Protonated Form)			
	direct H-transfer		direct H-transfer		mediated H-transfer (e^−^,H^+^,e^−^)	
	Estimated Free Energy of binding [kcal/mol]	N5(FAD)-O(NBA/AAI) distance [Å] ^a^	Estimated Free Energy of binding [kcal/mol]	N5(FAD)-O(NBA/AAI) distance [Å] ^a^	Estimated Free Energy of binding [kcal/mol]	OH(Y128)-O(NBA/AAI) distance [Å] ^b^
3-NBA	−5.7	3.7	−5.7	3.5	−6.2	3.1
AAI	−6.4	3.2	−6.3	3.2	−7.9	2.8

^a^, Distance between the oxygen in nitro group of AAI and that in nitro group of 3-NBA and nitrogen 5 of reduced FAD, see Figure 4 and Figure 5; ^b^, Distance between the oxygen in nitro group of AAI and in group of 3-NBA and OH group of Tyr128, see Figure 4 and Figure 5.

The orientations of the 3-NBA molecule in the active site is such that the nitro group is close to the hydrogen on N5 of the isoalloxazine ring of FAD (3.7 Å (Figure 4A) and 3.5 Å (Figure 4B) and Table 4). These spatial arrangements favor a hydride transfer to the nitro group of 3-NBA. A hydrogen bond between the OH group of Tyr128 and O7 of 3-NBA probably stabilizes this orientation, facilitating the reduction of 3-NBA (Figure 4) [52].

The orientations of the AAI molecule in the active site result in distances of the nitro group to the hydrogen on N5 of the isoalloxazine ring of reduced FAD of 3.2 Å in both forms (Figure 5A,B; Table 4), which are similar to those in 3-NBA binding to NQO1 and therefore are appropriate for a direct hydride transfer to the nitro group of this carcinogen.

However, the predicted binding affinities of these complexes are 1.5–1.7 kcal/mol weaker than those of another binding mode, which facilitates the alternative three-step (e^−^, H^+^, e^−^) reduction mechanism (Figure 5C, Table 4). The major factor making the direct hydride transfer more disadvantageous is probably the necessity of placing the nitro group close to N5 of FAD, which results in burying the neighboring carboxylic group of AAI deep into the hydrophobic binding pocket of NQO1. Moving of this highly polar group from solution into the hydrophobic environment of the non-polar binding pocket could therefore be a significant factor destabilizing the direct hydride transfer complex. AAI in the alternative three-step (e^−^, H^+^, e^−^) reduction complex, shown in Figure 5C, however, accommodates its carboxylic group near the access channel, thereby maximizing its contact with solvent. Hydroxy groups of Tyr128 and Tyr126 also contribute to the stabilization of this alternative AAI binding orientation through hydrogen bonding of oxygens of the nitro group (Figure 5). We believe this might be the major reason why the three-step (e^−^, H^+^, e^−^) reduction mechanism can play a role in reductive activation of AAI by NQO1.

This alternative three-step (e^−^, H^+^, e^−^) reduction mechanism is also possible for the reduction of 3-NBA by NQO1. However, the predicted binding affinities of complexes of the anionic and the protonated enol form with 3-NBA are only 0.5 kcal/mol weaker than those of another binding mode facilitating the e^−^, H^+^, e^−^ reduction mechanism (Figure 4C, Table 4). Therefore, this mechanism of 3-NBA reduction is less probable than that of AAI reduction.

The role of Tyr128 together with His161, as a general acid and proton donor during NQO1-catalyzed reduction of quinones, was proposed earlier [102], but only to support the hydride transfer from N5, or possibly N1. The (e^−^, H^+^, e^−^) reduction mechanism we propose would start from a protonated enol form of reduced FAD (stabilized by the enzyme), where electrons could be transferred directly via π-stacking interactions and the first proton from O2 (enol group of reduced FAD) is relayed through Tyr155, His161 and Tyr128 to the nitro-group of AAI, without direct hydride transfer from N5, N1 or O2.

### 4.2. Binding of 3-NBA and AAI to the Active Sites of CYP1A1 and 1A2

**Figure 6:**
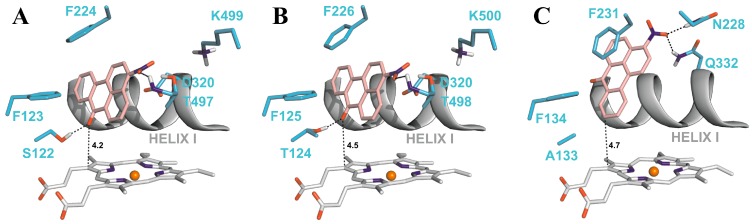
The most favorable binding orientations of 3-NBA docked into the active site of CYP1A1 (**A**), 1A2 (**B**) and 1B1 (**C**). Hydrogen bonds between 3-NBA and the amino acid residues in active site residues are rendered as dashed black lines. 3-NBA (pink), heme (grey) and side chains of important amino acid residues (cyan) are rendered as bold sticks; iron ions as orange spheres.

**Figure 7:**
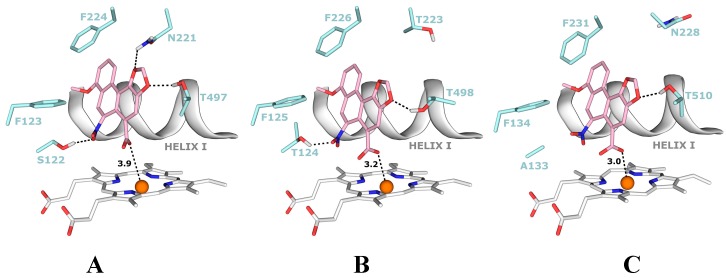
The most favorable binding orientations of AAI docked into the active site of CYP1A1 (**A**), 1A2 (**B**) and 1B1 (**C**). Hydrogen bonds between AAI and the amino acid residues in active site residues are rendered as dashed black lines. AAI (pink), heme (grey) and side chains of important amino acid residues (cyan) are rendered as bold sticks; iron ions as orange spheres.

Molecular modeling (*in silico* docking, employing soft-soft, flexible, docking procedure [52,69,73,90] was used also in studies to evaluate interactions of 3-NBA or AAI with the active site of some human CYPs, and to explain the high potency of CYP1A1 and 1A2 to reduce 3-NBA and AAI (Figure 6 and Figure 7). In addition, the results in these studies provided an explanation why mainly CYP1A1 and 1A2 are effective in the nitroreduction of these carcinogens, while their structurally similar analogue CYP1B1 is almost ineffective in catalyzing this reaction (Table 2) [77]. 3-NBA activation catalyzed by CYP1B1 reconstituted with POR was even lower than that by POR alone (Table 2). The direct hydride transfer that is typical for reduction of 3-NBA and AAI by NQO1 [52,69,73] is not applicable to the CYPs studied as they lack a suitable hydride donor group in their active sites. Therefore, only the stepwise reduction mechanism (e^−^, H^+^, e^−^, H^+^), which is an alternative for the reduction of AAI or 3-NBA by NQO1 (Section 2.3 and [69,73]) are applicable for CYPs.

Predicted binding free energies, distances between the 3-NBA ligand and a heme cofactor, and orientations of 3-NBA binding in the active sites of CYP1A1, 1A2 and 1B1 are similar. This is also true for the AAI ligand (Table 5, Figure 6 and Figure 7).

**Table 5:** The estimated binding free energies and distances between 3-NBA and AAI ligands and heme cofactor of CYPs for the best ranked complexes shown in Figure 6 and Figure 7. Data for AAI are taken from our previous work [90].

	CYP1A1		CYP1A2		CYP1B1	
	Estimated Free Energy of binding [kcal/mol]	3-NBA/AAI—heme distance [Å] ^a^	Estimated Free Energy of binding [kcal/mol]	3-NBA/AAI—heme distance [Å]^ a^	Estimated Free Energy of binding [kcal/mol]	3-NBA/AAI—heme distance [Å] ^a^
3-NBA	−8.04	4.2	−8.02	4.5	−7.73	4.7
AAI	−5.66	3.9	−5.81	3.2	−5.59	3.0

^a^, Distance between the heme cofactor and AAI or 3-NBA, see Figure 6 and Figure 7.

The most preferred orientations of 3-NBA in the active sites of all studied CYPs allows fast electron flow from the porphyrin ring of the heme cofactor. Predicted orientations of 3-NBA in the CYP1A1 and CYP1A2 active sites are virtually identical. The binding orientation of the nitro-group of 3-NBA in direct vicinity of a potential proton donor group, namely the hydroxyl groups of Thr497 and Thr498 in CYP1A1 and 1A2, respectively, allows reduction of this compound (Figure 6A,B). In contrast, the most preferred orientation of 3-NBA in the active site of CYP1B1 places the nitro-group close to amidic side chains of Asn228 and Gln332, which are inferior proton donors compared to the hydroxyl group of Thr497/Thr498 in CYP1A1/1A2.

The carboxylic group of AAI in the binding position is situated directly above the heme iron. This ligand orientation in CYP1A1/1A2 is further stabilized by two hydrogen bonds; one between an oxygen atom of the nitro-group of AAI and the hydroxyl group of Ser122/Thr124 and the second bond between an oxygen atom of the dioxolane ring of AAI and the hydroxyl group of Thr497/Thr498. However, for the complex of CYP1B1 with AAI any hydrogen bonding to the nitro group of AAI is prevented as Ser122/Thr124 residues in the CYP1A1/2 protein are replaced by the hydrophobic residue Ala133 in CYP1B1 (Figure 7A,B). Therefore, the hydroxyl group of Ser122/Thr124 residue, which is the only polar hydrogen placed close to the nitro group of AAI, is mechanistically important, providing a proton required for the stepwise reduction process [90].

## 5. Conclusions

The data summarized in this review, together with the results shown in Table 1 and Table 2, indicate that of the tested enzymes, NQO1 most efficiently activates 3-NBA and AAI to form DNA adducts *in vitro* and *in vivo*. Similar amounts of 3-NBA- and AAI-derived DNA adducts were formed during the reductive activation of these carcinogens by human NQO1 *in vitro*. This finding suggests that the two step reduction of these compounds to their ultimate reactive metabolite, *N*-OH-3-ABA from 3-NBA and *N*-hydroxyaristolactam I from AAI, proceeds with analogous efficiency. Further, they suggest that the reactivity of the ion formed from *N*-OH-3-ABA and the cyclic cation derived from *N*-hydroxyaristolactam I, reacting with nucleophilic centers of dG and dA bases in DNA should be similar. These suggestions were confirmed in studies utilizing thermodynamic approaches (Table 3) which also indicated similar mechanisms by which NQO1 catalyzes the reduction of 3-NBA and AAI. The results emphasize the importance of NQO1 in the genotoxicity of both carcinogens and demonstrate that the levels of NQO1 protein expression and its enzyme activity in human individuals are key determinants for a cancer risk of 3-NBA and AAI to humans.

The data in this review also shows the capabilities of human CYP1A1 and 1A2 to activate 3-NBA and AAI *in vitro* and *in vivo* and propose a mechanism for 3-NBA and AAI reduction reactions. The presence of the amino acid residues Ser or Thr that possess a polar hydrogen required for the stepwise reduction process in the active sites of both CYPs is necessary.

However, the efficacies of human CYP1A1/2 to reductively activate these carcinogens to their ultimate reactive intermediates are almost 24-fold lower than that of human NQO1 (Table 1 and Table 2). These results, together with findings showing similarities among the efficacies of the stepwise reduction of 3-NBA and AAI to their ultimate reactive intermediates (*N*-hydroxylated metabolites) and the reactivity of the ions derived from these intermediates binding to DNA, indicate that the lower efficiency of CYP1A1/2 compared to NQO1 to reductively activate both carcinogens might be caused by other factors. These are: (i) different affinities for NQO1 and CY1A1/2; (ii) the ease of the electron transport from the hydride donors (cofactors or amino acids in the active centers) to the nitro group; and/or (iii) the reaction kinetics catalyzed by both types of enzymes. The values of free energies of 3-NBA and AAI binding ranged from 5.7 to 8 kcal/mol, and are thus appropriate for reduction of both carcinogens. The predicted binding affinities of complexes of CYP1A1, CYPA2 and CYP1B1 with 3-NBA are 2.0–2.3 kcal/mol weaker than those of NQO1 with this carcinogen, which would facilitate a higher degree of reduction catalyzed by these CYPs than by NQO1. However, this is opposite to the results found experimentally. Hence, these findings indicate that the binding affinities are not the reason for the differences between the two enzymes in activation of these carcinogens to DNA adducts. The orientations of 3-NBA and AAI in the active sites of NQO1 and CYP1A1/2 showed shorter distances between the electron donors of NQO1 than those of CYP1A1/2, which might facilitate reduction by NQO1. Therefore, this feature might, at least partially, be responsible for the higher efficiency of NQO1 compared to CYP1A1/2 to reduce 3-NBA and AAI. However, these differences seem to be too low to explain an almost 24-fold difference in DNA adduct formation catalyzed by NQO1 compared to CYP1A1/2. Therefore, the kinetics of the reactions catalyzed by both enzymes seems to be an additional factor that contributes to the higher reductive activation of both carcinogens.

In conclusion, the combination of experimental and theoretical approaches was found useful to explain, at least to some extent, the NQO1- and CYP1A1/2-mediated reduction mechanisms of 3-NBA and AAI. Theoretical approaches need to be carefully interpreted, but can help to refine experimental findings.
